# Clinical Correlates and Drug Resistance in HIV-Infected and -Uninfected Pulmonary Tuberculosis Patients in South India

**DOI:** 10.4236/wja.2016.63013

**Published:** 2016-09-09

**Authors:** Chandy Sara, Heylen Elsa, Mishra Baijayanti, Ekstrand Maria Lennartsdotter

**Affiliations:** 1 Department of Medicine, St John's Medical College, Bangalore, India; 2 Center for AIDS Prevention Studies, University of California, San Francisco, USA; 3 Department of Microbiology, St John's Medical College, Bangalore, India; 4 St John's Research Institute, Bangalore, India

**Keywords:** Pulmonary TB, HIV, Anti-Tubercular Drug Resistance

## Abstract

**Objectives:**

To examine demographics, clinical correlates, sputum AFB (acid fast bacilli) smear grading DOTS (Directly Observed Therapy Short Course) uptake, and drug resistance in a cohort of newly-diagnosed, smear positive pulmonary tuberculosis (TB) patients with respect to HIV status at baseline, and compare smear conversion rates, side effects and mortality after two months.

**Design:**

A prospective study among 54 HIV positive and 41 HIV negative pulmonary TB patients. Data were collected via face-to-face interviews, review of medical records, and lab tests.

**Results:**

HIVTB co-infected patients, though more symptomatic at baseline, showed more improvement in their symptoms compared to HIV-uninfected TB patients at follow-up. The HIV co-infected group had more prevalent perceived side effects, and sputum smear positivity was marginally higher compared to the HIV negative group at follow-up. Mortality was higher among the HIV-infected group. Both groups had high rates of resistance to first-line anti-tubercular drugs, particularly isoniazid. There was no significant difference in the drug resistance patterns between the groups.

**Conclusions:**

Prompt initiation and provision of daily regimens of ATT (Anti-Tubercular treatment) along with ART (Anti-Retroviral treatment) via ART centers is urgently needed in India. As resistance to ART and/or ATT is directly linked to medication non-adherence, the use of counseling, regular reinforcement, early detection and appropriate intervention strategies to tackle this complex issue could help prevent premature mortality and development of resistance in HIV-TB co-infected patients. The high rate of isoniazid resistance might preclude its use in India as prophylaxis for latent TB in HIV infected persons as per the World Health Organization (WHO) guideline.

## 1. Introduction

India has the highest burden of tuberculosis (TB) in the world, with an estimated incidence of 2.2 million cases out of a global incidence of 9 million cases [1]. The prevalence of sputum smear-positive pulmonary TB is 2.27 per thousand compared to 5.05 per thousand of all forms of tuberculosis in India, and the average annual incidence of smear-positive cases is 84 per 100,000 [2]. HIV prevalence among incident TB patients is about 6 percent [3].

Through the Revised National TB Control Program (RNTCP), the Government of India provides free anti-tubercular treatment (ATT) known as Directly Observed Treatment, Short-course DOTS, a thrice weekly regimen delivered through state run and private sector providers [3]. Category 1 (CAT 1) DOTS is intended for new cases and consists of two months of intensive phase treatment with isoniazid (H), Rifampin (R), Pyrazinamide (Z), and Ethambutol (E), followed by four months of H and R. Category 2 (CAT 2) DOTS is for retreatment cases and has two months of HRZES (S = Streptomycin), one month of HRZE, and five months of HRE [3]. Patients who are adherent but do not achieve sputum smear conversion within two months following ATT initiation, require extended treatment [3] [4]. Sputum smear positivity after five months is considered to be failed treatment [3] [4]. Due to the enhanced survival benefits, it is recommended that HIV co-infected individuals receive anti-retroviral therapy (ART) regardless of CD4+ cell count soon after ATT initiation [5] [6].

Resistance to ATT has become a serious threat to TB control, with India harboring the second largest number of multi-drug resistant (resistance to H and R) TB (MDRTB) cases [7] [8]. The prevalence of MDRTB reflects the efficiency of TB management by health care providers from the public (RNTCP) and private sector as well as their collaborative efforts.

There are very few studies comparing the demographics, symptoms, DOTS uptake, side effects, and ATT adherence on pulmonary TB patients based on their HIV status [9] [10]. Also, given the slow growth of *mycobacterium* in culture (6 – 8 weeks), challenges with tracking reports are not being readily available, and the high cost, Drug Susceptibility Testing (DST) is not a routine practice. Only a few studies have compared sputum microscopy/culture conversion as an interim indicator for final treatment outcomes in relation to HIV status [11]–[14]. Some studies have shown that HIV infection is associated with low TB bacillary density in the sputum, higher culture conversion rates and a positive association between CD4T lymphocyte count and bacillary density [13] [14]. Other studies, especially those from India, have not shown a relationship between CD4-T lymphocyte count and bacillary density [11] [12] [15]. This current study aims to fill this gap in the literature by first examining these factors, including drug resistance patterns, in a cohort of newly diagnosed sputum Acid Fast Bacilli (AFB) smear positive pulmonary TB patients, both with HIV co-infection, and without HIV co-infection. Our second aim is to look at differences in symptoms, ATT side effects, smear conversion rates and mortality between these groups after two months.

## 2. Methods

### 2.1. Setting and Sample

Potential participants were referred to the study by staff of the pulmonary and general medicine outpatient departments, inpatient wards, the ART center, and the community HIV care center at St John's Medical College Hospital. These patients had clinical symptoms suggestive of pulmonary TB. Newly detected smear positive pulmonary TB patients from the referral sites and those who had definite radiological evidence of pulmonary TB pending smear results were included.

Written informed consent approved by the Institute Ethics Committee at St John's Medical College Hospital was obtained from all participants. For patients whose sputum tests were not already completed, researchers collected two sputum AFB smear samples, one spot and one early morning sample for testing [16]. Participants were considered eligible for the study if they were 18 years of age or older, willing to participate, and had one or more sputum smear samples test positive for AFB.

Participants were interviewed face-to-face for approximately one hour, and a sputum sample was tested for AFB culture and sensitivity. Those with unknown HIV status were screened for HIV infection per the National AIDS Control Organization (NACO) guidelines [17]. CD4 T cell counts were collected for all HIV-infected participants, if a test had not been conducted within the past month.

A one-month follow-up interview was conducted to examine improvement in symptoms, side effects and changes in ATT/ART medications, and to provide patients with AFB culture reports. Wherever possible, a two month post-intensive phase AFB smear report was collected from the RNTCP treatment cards, patient records, registers, or from the patients themselves.

Ninety-eight participants were enrolled in the study, of which three participants who were sputum smear and culture negative were removed; data of two participants whose AFB smears were negative but cultures subsequently grew *Mycobacterium Tuberculosis* were retained. Hence data analysis was done on 95 participants; 54 HIV-infected and 41-uninfected.

### 2.2. Measures

#### 2.2.1. Demographics

Participant age, gender, marital status, employment, education and residence were recorded.

#### 2.2.2. Symptoms and Side Effects

At baseline, participants indicated the symptoms they had experienced from a list of common TB symptoms (e.g. cough, fever, hemoptysis). At follow-up, a list of ATT side effects (e.g., nausea, vomiting, jaundice) was added. The number of endorsed symptoms and side effects were calculated.

#### 2.2.3. ATT/ART Medications

Participants were asked about medications consumed, whether they were on DOTS, had changes to ATT/ART, where their medications were procured and who paid for their drugs. ART adherence was assessed using a Visual Analogue Scale (VAS) [18]. For ATT they were asked daily/weekly details of medications consumed as the drugs were recently started.

#### 2.2.4. Blood, Sputum Collection and Drug Susceptibility Testing

We collected 5 ml of blood from each HIV positive participant for CD4 count (flow cytometry) testing. HIV negative participants were screened by Enzyme linked immunosorbent assay (ELISA) [17]. Sputum samples from each patient were collected in sterile containers and transported to the laboratory or stored at 4°C if a delay was anticipated. The sputum smear bacillary load was graded as scanty, 1+, 2+, 3+ per WHO/RNTCP [3] [19] guidelines. Samples for culture were decontaminated and inoculated to the Mycobacterium Growth Indicator tube (Micro-MGIT) for growth and identification. DST was performed for Rifampicin (40 μg/ml), Isoniazid (1 μg/ml), Ethambutol (2 μg/ml) and Streptomycin (4 μg/ml) using lyophilized drugs (Becton Dickinson Sparks MD).

### 2.3. Statistical Analysis

For demographic, clinical correlates and DST data, univariate descriptive statistics were calculated as appropriate. Differences between groups were analyzed by χ^2^, Fisher's exact test or logistic regression for categorical variables, and Mann-Whitney U-test or t-test for continuous variables, using SPSS Version 16 software. A two-tailed p-value <0.05 was considered statistically significant.

## 3. Results

### 3.1. Demographics

As shown in Table 1, nearly three-quarters of participants were male, and a majority was Hindu, married, and had children. The median age was 35 years. HIV-infected patients were less educated (p < 0.001) and had a lower median income than the uninfected group (Rs. 2000 vs. 4500, p = 0.012).

### 3.2. Clinical Correlates, DOTS Uptake and Past History of TB

As shown in Table 2, most patients presented with cough (96.8%), followed by fever (87.1%), weight loss (82.8%) and night sweats (77.4%). The HIV + TB group was more symptomatic than the TB only group (mean no. of symptoms 5.8 vs. 4.4, resp., p < 0.001). Overall DOTS uptake was 74.7%, with marginally (p = 0.074) fewer participants in the HIV infected group (50%) than in the uninfected group (73.2%) on CAT1 DOTS.

Nineteen percent of HIV infected participants and 9.8% of uninfected participants were onCAT2 DOTS, and 31.5% versus 17.1%, respectively, were on other daily regimens. Nearly one in three participants in either group had a previous history for TB (p = 0.873) with 15 HIV infected participants (27.8%) and 12 uninfected participants (29.3%) having previously been treated for TB.

### 3.3. HIV Infection and ART

The majority (n = 41/54, 76%) of the HIV infected patients reported getting tested for persistent illness, seven (13%) because their spouse tested positive, six (11.1%) on routine evaluation and one (1.9%) for high risk behavior. Twenty-one (38.9%) HIV infected participants were newly diagnosed, 24 (44.4%) were within five years (median: 1 year, IQR. 0 – 4 yrs). Forty participants (74.1%) were on ART at baseline. The majority were on d4t (stavudine) + 3TC (lamivudine) + NVP (nevirapine) or Efavirenz (57.5 %) and 37.5% were on AZT (zidovudine) + 3TC + NVP or EFV. The median CD4+ count of the HIV-infected group was 125/μl (IQR: 82 – 195). There was no relationship between CD4+ count and mortality or past history of TB.

### 3.4. Alcohol, Smoking and Co-Morbidities

Among the men, overall 37 (52.9%) were smokers and 41 (58.6%) used alcohol with no significant difference between the groups (p = 0.417 and p = 0.617). Nine (17%) of the HIV infected participants versus eight (20%) of the uninfected patients had a history of diabetes (p = 0.720). None had a history of drug abuse.

### 3.5. Symptoms and Side Effects at Follow-Up

Comparing Table 3 to Table 1, it shows that, in general, there was an improvement in symptoms at follow-up except for chest pain and hemoptysis. A comparison of the number of TB symptoms endorsed at baseline and 1 month follow-up, showed that the mean (SD) difference in symptoms endorsed in the HIV+ group was 0.63 (1.63) fewer symptoms, which was a significant decrease (p = 0.016), while the HIV− group had 0.30 (2.3) more symptoms at follow-up (p = 0.459). Perceived side effects at 1 month follow-up were significantly higher in the HIV+ group, on average, than in the HIV− group (mean 8.0 vs. 5.9, p = 0.042).

### 3.6. Changes in ART/ATT Medications

Among the HIV infected group, five participants who were ART naïve [20] were started on triple drug regimens and there were three NRTI substitutions. Ten participants on nevirapine were substituted with efavirenz. One stopped ATT; two were substituted with fluoroquinolones and a third on RZES daily changed to CAT1 DOTS in the HIV+ group, and one participant on HRZE daily to CAT2 DOTS in the HIV− group. Eight out of the 40 participants who were on ART at baseline reported ≤80% ART adherence in the past month. As participants had just started ATT at the time of assessment, the overall adherence rates were almost 100% for ATT.

### 3.7. 2 Month Post-Intensive Phase Follow up and Sputum Reports

Out of the 32 sputum smear samples available at 2 month follow up, nine (47.7%) from the HIV infected group and two (15.4%) from the -uninfected group remained positive (p = 0.13). By two months, from the data available on all the participants, eight(15.7%) of the TB+HIV group were known to be deceased, versus one (2.4%) in the TB only group, a significant difference in mortality (p = 0.04).

### 3.8. Resistance to ATT

Sputum samples were cultured for 77 participants (81%). The remaining 18 were not cultured due to technical problems (e.g. lack of sputum cups, lack of refrigeration in transport, n = 11), insufficient quantities of sputum (n = 2) and not returning for follow-up (n = 5). As shown in Table 4, 70.1% grew *Mycobacterium Tuberculosis*, 2.6% grew Mycobacterium other than TB, and there was no growth in the remaining 27.3%. Of the samples with available DST reports, 61.1% were resistant to Isoniazid, 31.5% to Rifampicin and MDRTB was observed in 24.5%. Eleven percent were resistant to all drugs tested (RHES). The odds of MDRTB were three times higher in the HIV-uninfected group than in the HIV group, controlling for history of TB (AOR = 3.14, 95% CI 0.74 – 13.38). However this difference was not statistically significant (p = 0.122).

## 4. Discussion

To our knowledge, this is the first time that symptoms, DOTS uptake, side effects, drug adherence, resistance and mortality were studied prospectively by HIV status in a cohort of exclusively smear positive pulmonary TB patients in India. We observed that the HIV+ group was more symptomatic for TB at baseline. Their symptoms significantly improved, but they had more adverse reactions compared to the HIV-uninfected group at one month follow-up. The higher average number of symptoms in HIV+ positive patients could be due to delayed reporting to the health care provider as symptoms are attributed to HIV disease, increased prevalence of smear negative pulmonary, extra-pulmonary or disseminated forms of TB [5] [21] [22] and diagnostic uncertainty due to ART-related side effects or other opportunistic infections. Drug interactions, shared drug toxicities of ATT/ART, or Immune Reconstitution Inflammatory Syndrome (IRIS) could have contributed to the large number of perceived side effects in the HIV co-infected group [5] [21] [22].

About three quarters of our respondents were on thrice weekly DOTS regimens, with marginally (p = 0.074) fewer of the HIV infected group on CAT1. The WHO guidelines suggest that HIV patients co-infected with TB should be treated with daily regimens in the initial intensive phase [6] [23] [24]. A recent meta-analysis found higher rates of ATT failure, and relapses or death due to TB if the initial intensive phase was administered thrice a week instead of daily, or if ART was not initiated [25]. This situation could be improved by linking the RNTCP with the National AIDS Control Program [3] to provide and dispense daily ATT along with ART for HIV co-infected participants in their ART center.

The HIV + TB group was less literate and had a smaller median income than the HIV-group, which mirrors the socio-demographics of HIV + TB patients in the rest of the world [26] [27]. Twenty percent of our HIV positive participants were non-adherent to ART. Most studies [28] [29] report increased non-adherence in co-infected patients with better adherence to ART than to ATT [28]. Though there were higher numbers of HIV infected smear positives at two month follow-up, the difference was not statistically significant. A recent study on smear conversion rates had only two HIV+ patients, both continuing to remain smear positive even at the end of the fifth month of treatment [30].

We observed very high rates of drug resistance overall, particularly with Isoniazid, which is consistent with other studies from Karnataka [7] [31]–[33] and elsewhere in India [34] [35] and has implications for the rationale of the current WHO recommendation of Isoniazid prophylaxis (IPT) for latent TB in the HIV infected [36] [37]. Furthermore, substantial emerging evidence challenges the preconceived paradigm of latent/active TB disease against the backdrop of HIV infection [38]. As observed world-wide [12] [22]–[24], there was a significant difference in mortality between the HIV-infected and -uninfected group at two months follow-up.

Our study has its limitations. The small size and the regional nature of the sample may not allow generalization of results to the whole country. The low number of two month follow-up sputum smear reports may have resulted in inadequate power to find significant differences in smear conversion rates between the groups.

## 5. Conclusion

We found that HIVTB co-infected patients were more symptomatic at baseline. They had more perceived side effects—possibly due to shared drug toxicities of ATT/ART, drug interactions or IRIS and a higher prevalence of sputum smear positivity and mortality at follow-up, compared to HIV uninfected TB participants. There was no significant difference in drug resistance patterns between the groups, with high rates of resistance to any first line anti-tubercular drug, particularly isoniazid. Prompt initiation and daily regimens of ATT along with ART, for HIV co-infected participants from their ART center will be a step forward to meeting the WHO guideline with respect to daily ATT. The very high rate of isoniazid resistance found in this and other studies across India [23] [31]–[35] might preclude its use as prophylaxis for latent TB in HIV infected individuals. As resistance to ART and/or ATT is directly linked to medication non-adherence, counseling regarding adherence to both regimens, regular reinforcement on follow-up, early detection and appropriate intervention strategies to tackle non-adherence could help prevent mortality and development of resistance to ATT in the HIVTB co-infected [39] [40].

## Figures and Tables

**Table 1:** Demographic characteristics of the sample.

	All		HIV + TB		TB only		

Characteristic	n	%	n	%	n	%	p-value^a^
Gender							0.521
Male	70	73.7	41	75.9	29	70.7	
Female	24	25.3	12	22.2	12	29.3	
Hijra	1	1.1	1	1.9	0	0.0	
Place of residence							0.304
Bangalore	41	43.2	30	55.6	22	53.7	
Other Karnataka	32	33.7	18	33.3	10	24.4	
Other state	22	23.2	6	11.1	9	22.0	
Education							0.000
None	19	20.0	12	22.2	7	17.1	
<4 years	18	18.9	17	31.5	1	2.4	
4 – 9 years	23	24.2	6	11.1	17	41.5	
≥10 years	35	36.8	19	35.2	16	39.0	
Marital status							0.639
Married	73	76.8	42	77.8	31	75.6	
Single	18	18.9	9	16.7	9	22.0	
Widowed	4	4.2	3	5.6	1	2.4	
Lives alone	18	18.9	14	25.9	4	9.8	0.046
Has children	63	66.3	38	70.4	25	61.0	0.337
Hindu	81	85.3	50	92.6	31	75.6	0.021
	median	IQR	median	IQR	median	IQR	
Age, years	35	30 – 43	35	32 – 41	35	25 – 53	0.889
Income, Rupees (if any, n = 85)	3000	1000 – 5000	2000	1000 – 4625	4500	1500 – 6000	0.012

a Difference between groups tested via chi-square test for categorical variables and Mann-Whitney U test for continuous variables.

**Table 2:** TB related variables.

	Available	All		HIV + TB		TB only		p-value^a^

Characteristic	n	n	%	n	%	n	%	
History of TB	95	27	28.4	15	27.8	12	29.3	0.873
TB symptoms:	93							
Cough		90	96.8	51	92.2	39	97.5	0.731
Fever		81	87.1	51	96.2	30	75.0	0.003
Night sweats		72	77.4	45	84.9	27	67.5	0.047
Shortness of breath		69	74.2	46	86.8	23	57.5	0.001
Chest pain		59	63.4	43	81.1	16	40.0	0.000
Blood in sputum		34	36.6	24	45.3	10	25.0	0.044
Weight loss		77	82.8	47	88.7	30	75.0	0.084
Symptoms, mean (SD)		5.2	(1.4)	5.8	(1.3)	4.4	(1.2)	0.000
TB regimen	95							0.074
DOTS cat 1		57	60.0	27	50.0	30	73.2	
DOTS cat 2		14	14.7	10	18.5	4	9.8	
Other		24	25.3	17	31.5	7	17.1	
Sputum AFB at baseline	95							0.638
Negative^b^		2	2.1	1	1.9	1	2.4	
Scanty		13	13.7	5	9.3	8	19.5	
1+		39	41.1	24	44.4	15	36.6	
2+		22	23.2	12	22.2	10	24.4	
3+		19	20.0	12	22.2	7	17.1	
2 mo. FU sputum smear positive	32	11	34.4	9	47.7	2	15.4	0.128

a Difference between groups tested via chi-square test for categorical variables, and t-test for continuous variables;

b These patients grew *Mycobacterium Tuberculosis* in culture.

**Table 3:** Side effects of TB Treatment after 1 month.

	All (n = 78)		HIV + TB (n = 44)		TB only (n = 34)		

	n	%	n	%	n	%	p-value^a^
Symptoms:							
Fever	63	80.8	37	84.1	26	76.5	0.563
Night sweats	51	65.4	25	56.8	26	76.5	0.094
Persistent cough	68	87.2	40	90.9	28	82.4	0.317
Expectoration	54	69.2	28	63.6	26	76.5	0.323
Blood in sputum	37	47.4	22	50.0	15	44.1	0.653
Difficulty breathing	49	62.8	30	68.2	19	55.9	0.346
Chest pain	52	66.7	35	79.5	17	50.0	0.008
Perceived side effects							
Jaundice	43	55.1	28	63.6	15	44.1	0.110
Skin rash	48	61.5	30	68.2	18	52.9	0.241
Tingling/numb fingers/toes	33	42.3	25	56.8	8	23.5	0.005
Nausea/vomiting	42	53.8	25	56.8	17	50.0	0.648
Stomach pain/cramps	44	56.4	28	63.6	16	47.1	0.171
Giddy/loss of balance	46	59.0	28	63.6	18	52.9	0.363
Decreased urine output	30	38.5	18	40.9	12	35.3	0.646
Joint pain	42	53.8	27	61.4	15	44.1	0.171
Headache	47	60.3	27	61.4	20	58.5	1.000
Swelling in feet	30	38.5	21	47.7	9	26.5	0.065
Swelling neck/groin/axilla	27	34.6	20	45.5	7	20.6	0.031
Difficulty seeing	24	30.8	17	38.6	7	20.6	0.137
Difficulty hearing	16	20.5	10	22.7	6	17.6	0.778
Weakness in limbs	23	29.5	17	38.6	6	17.6	0.050
Other	3	3.8	2	4.5	1	2.9	1.000
Total no. symptoms endorsed: mean (SD)	6.4	(4.4)	7.3	(4.3)	5.1	(4.4)	0.029

a Based on Fisher's exact test for frequencies and on t-test for mean.

**Table 4:** Resistance testing results.

	All		HIV + TB		TB only		

	n	%	n	%	n	%	p-value^a^
Cultured samples:	(n = 77)		(n = 40)		(n = 37)		0.284
No growth	21	27.3	14	35.0	7	18.9	
MOTT	2	2.6	1	2.5	1	2.7	
M-TB	54	70.1	25	62.5	29	78.4	
Resistant to:	(n = 54):		(n = 25)		(n = 29)		
Rifampicin (R)	17	31.5	7	28.0	10	34.5	0.770
Isoniazid (H)	33	61.1	14	56.0	19	65.5	0.579
Streptomycin (S)	15	27.8	7	28.0	8	27.6	1.000
Ethambutol (E)	20	37.0	10	40.0	10	34.5	0.780
H + R (=MDR)	13	24.1	4	16.0	9	31.0	0.223
H+R+S+E	6	11.1	2	8.0	4	13.8	0.675
Any drug	40	74.1	19	76.0	21	72.4	1.000

MDR, multi-drug resistance; MOTT, *Mycobacterium other than TB; M-TB, Mycobacterium Tuberculosis;*

a on Fisher's exact test for frequencies.

## References

[1] WHO (2015). Global Tuberculosis Report 2015.

[2] Chakraborty AK (2004). Epidemiology of Tuberculosis: Current Status in India. Indian Journal of Medical Research.

[3] Revised National Tuberculosis Control Programme (2005). Technical and Operational Guidelines for Tuberculosis Control.

[4] API Consensus Expert Committee (2006). API TB Consensus Guidelines 2006: Management of Pulmonary Tuberculosis, Extra-Pulmonary Tuberculosis and Tuberculosis in Special Situations. Journal of the Association of Physicians of India.

[5] Venkatesh KK, Swaminathan S, Andrews JR, Mayer KH (2011). Tuberculosis and HIV Co-Infection: Screening and Treatment Strategies. Drugs.

[6] WHO (2012). A Guide to Monitoring and Evaluation for Collaborative TB/HIV Activities.

[7] Gaude GS, Hattiholli J, Kumar P (2014). Risk Factors and Drug-Resistance Patterns among Pulmonary Tuberculosis Patients in Northern Karnataka Region, India. 2014. Nigerian Medical Journal.

[8] WHO (2008). Anti Tuberculosis Drug Resistance in the World: The WHO/IUATLD Global Project on Anti Tuberculosis Drug Resistance Surveillance. Fourth Global Report.

[9] Sharma SK, Manish Soneja KTP, Ranjan S (2014). Clinical Profile & Predictors of Poor Outcome of Adult HIV Tuberculosis Patients in a Tertiary Care Centre in North India. Indian Journal of Medical Research.

[10] Pradeep S (2011). The Situation of HIV/M. Tuberculosis Co-Infection in India. The Open Infectious Diseases Journal.

[11] Banu Rekha VV, Balasubramanian R, Swaminathan S (2007). Sputum Conversion at the end of Intensive Phase of Category-1 Regimen in the Treatment of Pulmonary Tuberculosis Patients with Diabetes Mellitus or HIV Infection: An Analysis of Risk Factors. Indian Journal of Medical Research.

[12] Swaminathan S, Deivanayagam CN, Rajasekaran S (2008). Long Term Follow up of HIV-Infected Patients with Tuberculosis Treated with 6-Month Intermittent Short Course Chemotherapy. The National Medical Journal of India.

[13] Mugusi F, Villamor E, Urassa W, Saathoff E, Bosch RJ, Fawzi WW (2006). HIV Co-Infection, CD4 Cell Counts and Clinical Correlates of Bacillary Density in Pulmonary Tuberculosis. International Journal of Tuberculosis and Lung Disease.

[14] Joloba ML, Johnson JL, Namale A (2000). Quantitative Sputum Bacillary Load during Rifampin-Containing Short Course Chemotherapy in Human Immunodeficiency Virus-Infected and Non-Infected Adults with Pulmonary Tuberculosis. International Journal of Tuberculosis and Lung Disease.

[15] Singhal S, Mahajan SN, Diwan SK, Gaidhane A, Quazi ZS (2011). Correlation of Sputum Smear Status with CD4 Count in Cases of Pulmonary Tuberculosis and HIV Co-Infected Patients: A Hospital Based Study in a Rural Area of Central India. Indian Journal of Tuberculosis.

[16] TB-C India Directorate General of Health Services, Ministry of Health and Family Welfare India (2009). Diagnosis of Smear Positive Pulmonary TB—New Guidelines, effective from 1 April 2009.

[17] NACO (2007). Guidelines for HIV Testing.

[18] Ekstrand ML, Chandy S, Heylen E, Steward W, Singh G (2010). Developing Useful Highly Active Antiretroviral Therapy Adherence Measures for India: The Prerana Study. Journal of Acquired Immune Deficiency Syndromes.

[19] WHO (1998). Laboratory Services in TB Control, Part II: Microscopy.

[20] AIDS Clinical Trials Group Trials Open for Enrollment.

[21] Dierberg KL, Chaisson RE (2013). HIV-Associated Tuberculosis: Update on Prevention and Treatment. Clinics in Chest Medicine.

[22] Mo P, Zhu Q, Teter C (2014). Prevalence Drug Induced Hepatotoxicity and Mortality among Patients Multi-Infected with HIV, Tuberculosis and Hepatitis Virus. International Journal of Infectious Diseases.

[23] Sethi S, Mewara A, Dhatwalia SK (2013). Prevalence of Multidrug Resistance in *Mycobacterium tuberculosis* Isolates from HIV Seropositive and Seronegative Patients with Pulmonary Tuberculosis in North India. BMC Infectious Diseases.

[24] Swaminathan S, Narendran G, Venkatesan P (2010). Efficacy of a 6-Month versus 9-Month Intermittent Treatment Regimen in HIV-Infected Patients with Tuberculosis: A Randomized Clinical Trial. American Journal of Respiratory and Critical Care Medicine.

[25] Khan FA, Minion J, Pai M, Royce S, Burman W, Harries AD (2010). Treatment of Active Tuberculosis in HIV-Coinfected Patients: A Systematic Review and Meta-Analysis. Clinical Infectious Diseases.

[26] Córdoba-Doña JA, Novalbos-Ruiz JP, Suárez-Farfante J, Andérica-Frías G, Escolar-Pujolar A (2012). Social Inequalities in HIV-TB and Non-HIV-TB Patients in Two Urban Areas in Southern Spain: Multilevel Analysis. International Journal of Tuberculosis and Lung Disease.

[27] Nibardo P-A, Mata-Marin JA, Gaytan-Martinez Jesus, Huerta-Garcia G, Acosta-Cazares B (2013). Clinical and Sociodemograzphic Risk Factors for Tuberculosis in Human Immunodeficiency Virus Infected Patients. American Journal of Infectious Diseases.

[28] Daftary A, Padayatchi N, O'Donnell M (2014). Preferential Adherence to Antiretroviral Therapy over Tuberculosis Treatment: A Qualitative Study of Drug-Resistant TB/HIV co-Infected Patients in South Africa. Global Public Health.

[29] Naidoo P, Peltzer K, Louw J, Matseke G, Mchunu G, Tutshana B (2013). Predictors of Tuberculosis (TB) and Antiretroviral (ARV) Medication Non-Adherence in Public Primary Care Patients in South Africa: A Cross Sectional Study. BMC Public Health.

[30] Parikh R, Nataraj G, Kanade S, Khatri V, Mehta P (2012). Time to Sputum Conversion in Smear Positive Pulmonary TB Patients on Category I DOTS and Factors Delaying It. Association of Physicians of India.

[31] Mishra B, Rockey SM, Gupta S, Srinivasa H, Muraleedharan S (2012). Multi-Drug-Resistant Tuberculosis: The Experience of an Urban Tertiary Care Hospital in South India Using Automated BACTEC 460 TB. Tropical Doctor.

[32] Burugina Nagaraja S, Satyanarayana S, Chadha SS (2011). How Do Patients Who Fail First-Line TB Treatment but Who Are Not Placed on an MDR-TB Regimen Fare in South India?. PLoS ONE.

[33] Nagaraja C, Shashibhushan BL, Sagar C, Asif M, Manjunath PH (2011). Resistance Pattern in Drug-Resistant Pulmonary Tuberculosis. Journal of Postgraduate Medicine.

[34] Kandi S, Prasad SV, Sagar Reddy PN (2013). Prevalence of Multidrug Resistance among Retreatment Pulmonary Tuberculosis Cases in a Tertiary Care Hospital, Hyderabad, India. Lung India.

[35] Magee MJ, Blumberg HM, Broz D (2012). Prevalence of Drug Resistant Tuberculosis among Patients at High-Risk for HIV Attending Outpatient Clinics in Delhi, India. Southeast Asian Journal of Tropical Medicine and Public Health.

[36] WHO (2011). Guidelines for Intensified Tuberculosis Case-Finding and Isoniazid Preventive Therapy for People Living with HIV in Resource-Constrained Settings.

[37] Gupta S, Abimbola T, Date A (2014). Cost-Effectiveness of the *Three I's* for *HIV*/*TB* and ART to Prevent TB among People Living with HIV. International Journal of Tuberculosis and Lung Disease.

[38] Lawn SD, Wood R, Wilkinson RJ (2011). Changing Concepts of “Latent Tuberculosis Infection” in Patients Living with HIV Infection. Clinical and Developmental Immunology.

[39] Nieuwlaat R, Wilczynski N, Navarro T, Hobson N, Jeffery R, Keepanasseril A, Agoritsas T, Mistry N, Iorio A, Jack S, Sivaramalingam B, Iserman E, Mustafa RA, Jedraszewski D, Cotoi C, Haynes RB (2013). Interventions for Enhancing Medication Adherence. Cochrane Database of Systematic Reviews.

[40] Rueda S, Park-Wyllie LY, Bayoumi A, Tynan A-M, Antoniou T, Rourke S, Glazier R (2006). Patient Support and Education for Promoting Adherence to Highly Active Antiretroviral Therapy for HIV/AIDS. Cochrane Database of Systematic Reviews.

